# One Health and EcoHealth in Ontario: a qualitative study exploring how holistic and integrative approaches are shaping public health practice in Ontario

**DOI:** 10.1186/1471-2458-12-358

**Published:** 2012-05-16

**Authors:** Zee Leung, Dean Middleton, Karen Morrison

**Affiliations:** 1 Department of Population Medicine, Ontario Veterinary College, University of Guelph, Guelph, Canada; 2 Public Health Ontario, Toronto, Canada

**Keywords:** Public health practice, One Health, EcoHealth, Governance, Sustainability, Cross-sectoral partnerships

## Abstract

**Background:**

There is a growing recognition that many public health issues are complex and can be best understood by examining the relationship between human health and the health of the ecosystems in which people live. Two approaches, One Health and Ecosystem Approaches to Health (EcoHealth), can help us to better understand these intricate and complex connections, and appear to hold great promise for tackling many modern public health dilemmas. Although both One Health and EcoHealth have garnered recognition from numerous health bodies in Canada and abroad, there is still a need to better understand how these approaches are shaping the practice of public health in Ontario.

The purpose of this study was to characterize how public health actors in Ontario are influenced by the holistic principles which underlie One Health and EcoHealth, and to identify important lessons from their experiences.

**Methods:**

Ten semi-structured interviews were conducted with ten participants from the public health sphere in Ontario. Participants encompassed diverse perspectives including infectious disease, food systems, urban agriculture, and environmental health. Interviews were recorded, transcribed and analyzed using qualitative content analysis to identify major themes and patterns.

**Results:**

Four major themes emerged from the interviews: the importance of connecting human health with the environment; the role of governance in promoting these ideas; the value of partnerships and collaborations in public health practice; and the challenge of operationalizing holistic approaches to public health. Overall study participants were found to be heavily influenced by concepts couched in EcoHealth and One Health literature, despite a lack of familiarity with these fields.

**Conclusions:**

Although One Health and EcoHealth are lesser known approaches in the public health sphere, their holistic and systems-based principles were found to influence the thoughts, values and experiences of public health actors interviewed in this study. This study also highlights the critical role of governance and partnerships in facilitating a holistic approach to health. Further research on governance and partnership models, as well as systems-based organizational working practices, is needed to close the gap between One Health and EcoHealth theory and public health practice.

## Background

There is a growing recognition that many issues in public health are highly complex and can be best understood through a systems lens, one that connects the health and well-being of humans with the larger ecosystem. For example, shifting our focus towards the environment has helped us to connect the emergence of a zoonotic virus in East Asia with human encroachment upon wild areas, or the *E. coli* outbreak in Walkerton with climatic conditions and an overburdened municipal water authority [1].

Two approaches, One Health and Ecosystem Approaches to Health, can help us to better understand these intricate and complex connections. Although One Health and EcoHealth are closely related, they have arisen from different traditions and can also be applied in very different trajectories. One Health examines issues at the intersection of human, animal and environmental health, and is “dedicated to improving the lives of all species - human and animal - through the integration of human medicine, veterinary medicine and environmental science” [2]. This approach can be traced to early thinkers such as William Osler, Calvin Schwabe and Rudolf Virchow, who recognized the close relationship between the worlds of human and veterinary medicine [3]. Conservationists further contributed to this movement through the “Manhattan Principles” which links the health of humans and domestic animals with the health of ecosystems [4]. Consequently, the One Health movement places a strong focus on areas such as zoonotic disease epidemiology and surveillance, biomedical research in comparative medicine and greater collaboration between the medical, veterinary, public health and environmental science communities [2].

Ecosystem Approaches to Health (EcoHealth) has developed within the same family of holistic approaches as One Health. In comparison to One Health, EcoHealth adopts a much broader perspective of health and is informed by diverse fields including natural resource management, health geography, systems sciences, philosophy and public health. EcoHealth has also been heavily shaped by the sustainable development movement of the 1980s. The seminal Brundtland Report of 1987 espoused key principles such as social justice, participation, and equity – both inter-generational and intra-generational [5]. These principles and the holistic spirit of the Brundtland Report, inform much of current EcoHealth thinking and practice.

One of the first documented uses of an EcoHealth approach was by the Great Lakes Research Advisory Board in 1978. Their report to the International Joint Commission drew attention to the diverse and complex interactions between the Great lakes and its surrounding social and ecological systems [6]. Additional research in ecological systems and complex systems theory, have led to a greater understanding of adaptive management, resilience and nested systems [7] – all important concepts in EcoHealth.

Thus a diversity of concepts, frameworks and philosophies has found a home within the EcoHealth community. For EcoHealth practitioners, the “integration of many diverse and often differing perspectives” can lead to a richer understanding of health in socio-ecological systems [8].

However the immense scope of this approach can create a challenge in arriving at a singular, concise definition of EcoHealth. One definition offered by Charron [9], sees EcoHealth as the recognition that “health and well-being are the result of complex and dynamic interactions between determinants, and between people, social and economic conditions, and ecosystems” and that EcoHealth “goes beyond prevailing biomedical or epidemiological approaches to health research”.

One Health, EcoHealth and related holistic perspectives about health, appear to hold great promise for tackling many modern public health dilemmas. Not surprisingly, a number of major health bodies are exploring these approaches, including the International Development Research Centre (IDRC) [10], Public Health Agency of Canada [11], the World Health Organization, the United Nations System Influenza Coordination, and the Food and Agriculture Organization [12]. In addition, EcoHealth was recognized as a 2010 Population and Public Health Research Milestone by the Canadian Institutes of Health Research, the Institute of Population and Public Health, and the Canadian Public Health Association. In their awards profile of EcoHealth, Webb et al. [13] declare that these approaches

"“represent an important and timely paradigm shift. Simultaneously and systematically embracing environmental sustainability, transdisciplinarity, social justice and gender equity, and stakeholder participation provides a pathway, not only to understand complex problems in public health but also to translate that knowledge into effective policy and action at the local, national and global levels.”"

Despite this significant amount of domestic and international support, recognition of One Health and EcoHealth in the public health sphere is still relatively recent. This is in contrast to the long history of the holistic philosophies which actually underlie these approaches - within public health as well as other disciplines such as environmental management and international development studies. Still, clues to the influence of these approaches can be found within the ever-expanding scope of public health practice. For instance, issues around the built environment, food systems, zoonotic disease and climate change, are examples of ‘wicked problems’ [14]–complex challenges for which there are no simple uni-disciplinary solutions. Understanding problems of this nature necessitates involving a diverse group of public health actors including individuals, communities, organizations, professional bodies, and institutions from the state, civil society and private domains.

Studying the work of this diverse group of public health actors might reveal a broad acceptance and level of understanding of the holistic principles which are foundational to One Health and EcoHealth. Moreover lessons learned from public health actors in these domains could hold valuable insights into how these philosophies can be translated into tangible public health programs and practices.

The goal of this study is to characterize: a) how actors in the public health sphere are influenced by the holistic principles underlying One Health and EcoHealth; b) what barriers and/or support systems these actors encountered in applying these holistic ideas; and c) what lessons could be learned from their experiences. Understanding and reflecting upon the experiences of public health actors in Ontario may hold important lessons as to how we can close the gap between One Health and EcoHealth theory and public health practice.

## Methods

### Study setting

Located in the central-east region of Canada, Ontario is the most populous province or territory in the country. The province’s 13 million citizens mainly reside along the Great Lakes region in Southern Ontario. Public health responsibility formally resides among 36 public health units distributed across the province. The responsibilities and activities of these health units are developed around the Ontario Public Health Standards [15] and Protocols, a series of guidelines published by the provincial Ministry of Health and Long-term Care. These guiding documents outline expectations for the province’s health units in meeting the mandatory health programs and services described in the Health Protection and Promotion Act [16].

### Development of semi-structured interview

A semi-structured interview was used to better understand the experiences of public health actors with integrative approaches around health and the environment. This interview was developed around three key areas:

1) Organizational identity and network (addressing ideas around fitting into the larger public health system, and on engaging stakeholders and communities);

2) Human health and the environment (trying to understand how the relationship between health and the environment is understood by participants, how it is reflected in their programming, and what support systems and/or barriers they have encountered); and

3) Organizational working practices (addressing concepts such as the identification and prioritization of public health problems, knowledge translation and knowledge exchange).

After preliminary development, the interview was evaluated and refined through three rounds of pre-testing. The questions used in the interview guide are included in Table 1.

**Table 1:** Interview Guide

**Q.**	**Main Questions**	**Additional Questions**	**Clarifying Questions / Prompts**
1.	Describe your organization, its mandate, and responsibilities		
2.	Who (what population/community) is the focus of your activities?		
3.	Is there a larger system that your community is a part of?	How does the work of your organization fit into this larger system?	What are the links? How do they connect in a public health context?
4.	Who are the stakeholders in your community?	How do you identify these stakeholders?	
5.	Are there unique or marginalized groups within your community?		
6.	Are they represented in your programming? Why or why not?		
7.	How does your organization work with stakeholders?	Are stakeholders directly involved with any aspect of your work?	Communication, direct involvement, program development, conducting research/running programs, analysis/evaluation or dissemination
8.	Are your stakeholders directly represented within your organization?	If so, how?	Structurally (peer researchers, board membership, etc.) or through your programs (directly engage stakeholders, represent interests)?
9.	Are there any barriers to working with stakeholders?	How does your organization try to overcome these barriers?	
10.	How do you define “health”?	How do you define “health” in your community?	
		Are there other definitions of health in your community?	
11.	How do you define the environment of your community?	What are/Are there links between your community and the environment?	
12.	Do you believe that the health of your community is linked to its environment?	If so, how?	
		Is this reflected in your programming?	
13.	How do you define a “healthy” environment?		
14.	Do you believe that the health of your community is linked to the health of its environment?	If so, how?	
15.	Is this reflected in your programming?	Why or why not? If not, what are the barriers to doing so?	
16.	How do you measure health (of your community and the environment)?		
17.	How do you know if you’ve improved the health of your community?	Their environment?	
18.	Are your perspectives on the connection between environment and the health of your community, shared by your partners?	Your community?	
19.	If so, how are your programs/policies supported by your partners? By your community?		
20.	If not, what are the barriers towards a shared understanding?		Can you elaborate or give specific examples?
21.	In the context of the health and the environment, is there an individual / policy / program / organization that has shaped the practices of your organization?		
22.	Can you identify a model of good public health practice with respect to health and the environment?		
23.	How are public health problems first identified by your organization?	How are they prioritized?	
24.	When developing your programs and policies, what types of evidence do you collect?	How do you collect this evidence?	For example, scientific/formal literature, expert-driven, community level, local or individual knowledge
		How do you prioritize evidence?	
25.	What are the challenges to integrating evidence into your programming?		
26.	Who is involved in developing programs and policies?		Individuals? A team approach? If a team, who would typically make up this team?
27.	Do team members have different perspectives on the links between human health and the environment? Do you incorporate perspectives from outside the organization? If so, how?		From individuals, from the community, other groups?
28.	How do you reconcile conflicting perspectives or pieces of evidence?		
29.	How do you disseminate or share your work? With whom is your work shared?		
30.	Are your programs and policies adaptive? If so, how?		Responsive to changing environments?
31.	At what scale (temporal or spatial or otherwise) are your policies and programs developed?		

### Identification and recruitment of participants

Participants in this study were identified through a mixture of snowball sampling and convenience sampling. The bulk of the study participants were identified from the 2011 Ontario Public Health Conference, as well as Public Health Ontario’s Public Health Research, Education and Development project. Finally the OPHLA Custom Search Engine [17] for searching public health units was also used. A variety of search terms such as “ecosystem AND health”, “holistic AND health”, “environment AND health”, “one health”, “ecohealth” and “one world one health” were entered into this search engine.

Using this search strategy, we identified participants dealing with complex public health issues at the intersection of health and the environment.

Throughout this process, there was a strong commitment towards seeking out stories and lessons from outside the traditional public health sphere. Ten key informants from within the broad public health sphere were recruited into this study. Participants were not selected by any prior knowledge or awareness of either One Health or EcoHealth. Rather, participants were selected if the study authors believed their experiences could hold valuable insights around health and the environment.

The ten participants included in this study were recruited from a number of diverse sectors, jurisdictions and fields. These participants represented a wide range of perspectives including infectious and zoonotic disease, food systems, urban agriculture, environmental health and community health. Four participants were recruited from local public health units, two were from municipal organizations, and the remaining four came from a provincial agency, a federal agency, a health research centre and a First Nations health advisory group.

### Participant interviews

After recruitment, each participant was administered a 60 minute semi-structured interview using the previously described interview guide. Interviews were either conducted in person or over the phone. Each interview was digitally recorded and transcribed. Transcripts were sent to the study participants for review and editing.

### Qualitative content analysis

Interview transcripts went through an iterative coding process using a word processing software (Microsoft Word, Microsoft Corporation, Redmond, WA). Over three iterations, a coding list was developed and refined, while text references from the transcripts were simultaneously analyzed and coded appropriately (Table 2). Finally content analysis was performed on coded transcripts using qualitative data analysis software (NVivo 9, QSR International Pty Ltd., Australia). Qualitative content analysis was wholly performed by the primary author of this study.

**Table 2:** Coding guide and sample coded text

**Code**	**Definition**	**Sample Text**
Barriers	Challenges or obstacles encountered by the participant	*“I think it can be overwhelming to think about how much you have to bring in to solving a problem”*
Crossing Sectors and Silos	Practices or approaches that cross boundaries/jurisdictions of sectors, organizations, or other silos	*“I would say that each project has a core group of stakeholders – that group of stakeholders is rarely within the context of one discipline or one sector”*
Environment	Discussion of the environment - defining it, environmental health issues, its connection to public health	“*It’s kind of a systems focus on how changing one piece changes all of those nested hierarchies of environments that are sitting within each other”*
Equity	Discussion of issues of equity especially around health	*“Photovoice was a really powerful way of hearing the most vulnerable within our community. And really engaging them as well. Not just hearing them but helping them to develop skills and advocacy”*
Governance	Discussion of current and possible models of governance, and their implications to public health	*“So there are a lot of times, as a community our hands are tied […] So sometimes we can’t do anything because of bureaucracy”*
Health	Discussion of health - defining it, important health issues	*“if we’re focusing on human health and well-being, often we forget about broader ecosystem and ecological issues […] sustainability issues, energy”*
Important Need	An identified need or important issue for public health practice	*“a real recognition that we need to reach out to stakeholders and other jurisdictions if we have a broader systems based approach and learn how to work with them and learn how to bring them into the tent“*
Indicators	Discussion around measurement tools or indicators for health	*“People across North America have been struggling with this idea about how do you develop indicators for a so-called healthy food system or community food security*”
Methods	Discussion of innovative or interesting approaches in public health	*“deliberative policy analysis […] it’s the idea that through reflection and deliberation on policy issues, we actually develop some policy learning and that’s what can move change forward”*
Participatory Approaches	Discussion of participatory approaches in public health practice. Also includes peer research, community-based research, community engagement and community involvement.	*“Community engagement - how do we have those conversations with community members for them to understand and be a part of this process?”*
Perspectives, Context	Discussion of other perspectives or other contexts, and their role in public health.	*“What we’re really trying to do is to have those sectors that we haven’t traditionally partnered with. I think traditionally […] we’ve had like-minded people at the table and so those people who have a different point of view from us, not necessarily are we hearing their voices”*
Support System	Support systems or facilitators for the work of the participant.	*“There’s been lots of support definitely within the community”*
Sustainability	Discussion of sustainability, self-sustaining systems, or visioning for the future.	*“So what are we doing to our biophysical environment both in terms of resource use, resource extraction? So how much are we consuming that we are taking away from future generations?”*
Systems Thinking	Consideration of smaller/larger systems, drawing connections, recognizing nested scales	*“a broader understanding that if you’re thinking from an ecosystem perspective, if you’re changing something in any of those environments, it’s going to affect what’s happening in the others”*
Inter- or Transdisciplinary Practices	Practices which engage multiple disciplines and/or transcends disciplinary thinking to explore different types of knowledge and ways of knowing	*“It’s a wide variety of evidence. It’s qualitative and quantitative. It’s peer-reviewed literature but also policy literature […], it includes stakeholder perspectives on the issue, it includes gathering evidence about the experience of jurisdictions*“

### Confidentiality

All data collected from participants were kept anonymous. An alpha-numeric coding system was applied to study data and stored under password-protected files at Public Health Ontario.

### Ethics approval

This study was conducted as part of the Public Health Practicum course for the Master of Public Health program at the University of Guelph. This study was approved by the Research Ethics Board of the University of Guelph (Protocol #11MY036) and was also assessed to fall under the research mandate of Public Health Ontario. Additional ethics approval by research ethics boards associated with any specific study participants were also received.

## Results and discussion

During the interview sessions, participants reflected upon a number of concepts around holistic and integrative practices surrounding health, the environment, and systems thinking. Participants were also able to ground these discussions by drawing connections to their experiences in public health and how these ideas may have shaped their work. From these discussions, ten transcripts totalling 48,398 words were coded under fifteen different codes (Table 2).

Following categorization under these 15 codes, content analysis was performed in interview data. This analysis revealed a number of insights arising from all of these discussions which could be grouped under four major themes:

1. Health, the environment and systems

2. The role of governance

3. Making connections, forming bridges and closing gaps

4. Challenging next steps

The following section describes the ideas and discussions captured under these themes, and examines their relationship to core principles of One Health and EcoHealth practice.

1. On health, the environment and systemsBoth One Health and EcoHealth critically re-examine and redefine our conceptualizations of health and the environment, especially through a broad systems lens. These approaches encourage reflective thinking that greatly expands on the traditional biomedical definition of health. In doing so, we begin to consider ideas around the health of animals and ecosystems, sustainability (in all its diverse forms), transdisciplinarity and other knowledge cultures, equity and social justice [13]. Further exploration of these ideas inevitably leads to reflections on the types of values embedded in our definitions of health, and encourages asking questions such as “Whose idea of health?”, “Health for whom?” and “Health for all or health for some?”.Similarly, our notion of the environment begins to look a little bit differently under a holistic systems lens. This is perhaps where EcoHealth most significantly distinguishes itself from environmental health, an important field in its own right. Where the latter focuses heavily on the traditional biophysical definition of the environment, a holistic systems discourse includes, among others, socioecological, economic, cultural and policy-framed conceptualizations of the environment. These are all complex academic concepts grounded in philosophy, epistemology, ethics and the systems sciences, and as such, do not lend themselves easily towards providing concise and neatly packaged concepts or definitions. Indeed, the challenge of developing an understanding of these seemingly simple concepts such as health and the environment was evident in many of the interviews. When asked to define health, many participants struggled to provide a clear definition without any afterthoughts or concessions.Similarly, our notion of the environment begins to look a little bit differently under a holistic systems lens. This is perhaps where EcoHealth most significantly distinguishes itself from environmental health, an important field in its own right. Where the latter focuses heavily on the traditional biophysical definition of the environment, a holistic systems discourse includes, among others, socioecological, economic, cultural and policy-framed conceptualizations of the environment. These are all complex academic concepts grounded in philosophy, epistemology, ethics and the systems sciences, and as such, do not lend themselves easily towards providing concise and neatly packaged concepts or definitions. Indeed, the challenge of developing an understanding of these seemingly simple concepts such as health and the environment was evident in many of the interviews. When asked to define health, many participants struggled to provide a clear definition without any afterthoughts or concessions.The definition of health as conceived by the World Health Organization [18] was a common starting point for many participants. However participants expanded on this definition to include discussions of equity, access to resources, socioeconomic disparities, community health and economic health.Additionally, the majority of the participants found the traditional biomedical definition of health to be lacking on a number of fronts. For example one participant observed that:Some participants did away with the idea that health had to be a singular, definable concept and importantly, discussed the role of context – an argument heavily couched in EcoHealth and One Health thinking. EcoHealth researchers often stress the importance of perspective and context in framing and understanding health and disease. These scientists grapple with the difficulty of answering deceptively simple questions when working in systems such as: “how do we define the ‘patient’ – not just its boundaries but also its internal dynamics” [19]? For example, Waltner-Toews et al. use an example of a simple patch of grass to illustrate this challenge. [19] In trying to define the health of this ‘patient’, Waltner-Toews et al. first struggled to understand the purpose and value of this plot of land: Was the plot of land more important as fertile land for agricultural development or was it a vibrant space for community recreational activities [19]? Perhaps this patch of green space represented something else entirely.Furthermore, expanding upon traditional biomedical concepts to include other perspectives, leads to the emergence of even more challenging issues: Who claims ownership or bears responsibility for the system? How do we even define what the problem is and consequently, how do we define the solution?The answers to these questions fundamentally dictate our understanding of our ‘patient’, its state of health and subsequently, our response to this system. As eloquently stated by Waltner-Toews et al. [19], “perspective changes everything”.These ideas were evident in several interviews with participants describing health as a constantly shifting and highly contextual concept. Health was not seen as something that could be imported unchanged across disciplines, communities or time scales. Furthermore, a commonly expressed point was the importance of adaptability and being able to shift one’s perspective on health to adapt to local contexts and understandings.The challenges of definitions and the importance of contextualization also emerged during discussions around the environment. Multiple concepts of the environment were discussed during the interviews, with participants discussing the built, social, economic, urban, policy and food environment. Moreover, one participant stressed the importance of working with the community to better understand how other groups in the population understood and valued the environment. This type of community engagement was stressed to be a core part of the participant’s programming.Some participants also viewed environments as existing within nested systems of other environments. Using a holistic lens as a contextual tool helped participants to navigate these complex systems and understand the multiple downstream and upstream connections between the health of their community and the multiple conceptualizations of the environment. For example, in considering the role of food systems in their community, one participant “*saw food as having a direct effect on the local economy, the local environment and our local health of the community in the sense of its connectivity and social capital and relationships”*. In the words of another participant, “*It’s kind of a systems focus on how changing one piece changes all of those nested hierarchies of environments that are sitting within each other”*.The inextricable links and interconnections between health and the environment was another common discussion which emerged from the interviews. Certainly this is not a new idea. Within the environmental health field, environmental factors have been long documented as causative agents of disease and illness. What is perhaps unique about these discussions however is that participants described the environment as more than just a determinant for health. Rather, participants viewed health and the environment as fundamental and necessary components of each other. For example, in discussing this connection, one participant stated, *“I think they’re completely integrated. I mean, you can’t get out of it”.* For another participant, the environment was an essential component of their definition of health: *“you have to look at the environment, how healthy is the air? Is it good to breathe? And how much can you get out and about?…That’s all to me, part of health”.* Finally, one participant defined a healthy environment as “*one that does not negatively affect the physical, mental or social well-being of people…and which even promotes physical, social or mental well-being, good health”*.These ideas which link the health of people with the environment were found to be significant not only from an academic standpoint, but also a practical perspective because participants could view holistic and sustainable practices as creating a “double-dividend” [20] for both health and for the environment. For one participant, this was evident in their discussion on the importance of the built environment for health:Although many of these holistic reflections on health were seen as intuitive by the participants in this study, they were not commonly shared among their partners or other actors within the larger public health system. Many participants identified these incongruent perspectives as major obstacles towards healthy collaboration with their stakeholders.This lack of shared understanding was identified across a range of stakeholders, from bureaucrats (*“we’ve been consistently educating the government, the bureaucracy, in terms of where we’re coming from”),* to the general public (“*yeah people* [the public] *don’t even get it. And people you’d think would get it. And if you think, if they’re not getting it… how are we ever going to sell this*”), and even to other actors within the public health sphere (“*It’s not their mandate. They don’t have to think that way, it’s not required*”).Overall holistic and systemic perspectives about health and the environment were commonly discussed by participants. Participants’ perspectives were diverse and far-reaching, with health and the environment commonly seen as contextual, shifting, nebulous and inextricably linked with each other. Applying a holistic or systems lens towards these concepts allowed some participants to make sense of their inherent complexity and to better understand their importance in various contexts at the community level. Finally, participants identified major challenges in creating a shared understanding about health, the environment and systems thinking. These barriers speak to larger issues of working with different partners and stakeholders. Thus various governance structures and models of stakeholder collaboration emerged as a key theme from the interviews. These discussions are profiled in the next section.

2. The Role of GovernanceThere is no single way to teach EcoHealth or One Health, or to understand how these may be practiced - their pluralistic nature creates an inclusive space for multiple perspectives and approaches. In considering the role of governance, however, one useful heuristic may be to conceptualize these holistic practices as having two main arms or branches: the first considers the complex, systems-based interconnections between health and the larger environment, and the myriad issues which emerge from that line of thinking; the second focuses on the *process* by which these connections are understood and acted upon. It is in this second branch that models of governance and working with different stakeholders becomes crucial to the conversation.This idea is echoed in the fact that governance and engaging stakeholders in the larger system were major themes discussed by study participants. Interestingly, governance was seen as an important structural force that could have both stifling and supportive effects towards holistic approaches to public health practice.Governance was found to act as a barrier towards holistic practices in a number of ways. For example, participants identified navigating the complex web of mandates and jurisdictions as a major challenge towards working within a larger system of stakeholders and partners:When considering how to work with multiple stakeholders on a particular issue, there was a great deal of confusion and uncertainty on a number of different levels: What were the mandates and responsibilities of the stakeholders involved? How did their mandates fit with the issue in question? Whose responsibility did that issue fall under the direct purview of? Who needed to assume responsibility?And finally, how does one reconcile issues of overlapping or conflicting mandates and jurisdictions?Better methods of communication were identified as one way to improve collaboration between multiple stakeholders and overcome some of these jurisdictional issues. For example, one participant stressed the need to learn each other’s ‘languages’ – how to speak and interact with different groups, disciplines, sectors and knowledge cultures. Doing so could lead to a shared understanding of each others’ perspectives and their individual roles in the larger system.Challenging relationships with management and other political forces were identified as another important obstacle faced by participants. This was manifested through: communication barriers between scientists and decision makers (*“as a scientist, it becomes really hard, you know a lot of the work that we do to inform policy has to be put into a very politic way to move forward”);* political or jurisdictional barriers limiting the powers of public health practitioners (“*So sometimes we can’t do anything because of bureaucracy*”); and finally, the difficulties in negotiating the role of public health practitioners in policy development while under a governing political framework (“*but we are public servants, so we are going to help facilitate that process, we can’t articulate that policy ourselves*”).Working in this highly political environment was seen as challenging to one participant’s ideals of value-neutral scientific objectivity.By contrast, other participants were much more comfortable with this intersection between science and politics. Organizational mandates and working practices were thought to be greatly strengthened by the influence of leaders and champions of particular causes. Strong political voices could help to advance ideas and push forward agendas on holistic working practices. Interestingly, one participant even planned a healthy communities advocacy campaign around a local political election. This innovative form of community engagement was a way to encourage community members to connect the health of their community with the local political environment.Despite creating some major barriers, models of governance were also found to be an important support system for several participants. For example, one participant said of a particular governance arrangement:Additionally, top-down governance mechanisms were seen as one way to promote holistic and systems perspectives around health and the environment. One participant pointed to the importance of the Ottawa Charter as “*a guiding model for everything that we do”*. Another participant discussed how the Ontario Public Health Program Standards have brought issues such as air quality, climate change and built environment to the forefront of the public health discourse:The emergence of governance as an important theme arising from these key informant interviews is not surprising. Governance as a driving force for the health and sustainability of natural systems has long been a core tenet of the natural resource management sphere. This holds true when shifting our perspective towards the intersection of the environment with human systems, also known as socio-ecological systems. In studying watersheds as a common point of conversation between health systems, ecosystems and social systems, Parkes et al. [20] illustrated the value of applying a governance lens to best link these multiple perspectives. By doing so, Parkes et al. [20] were able to “compare and contrast a range of perspectives, while highlighting gaps in knowledge and understanding…and identifying new opportunities for integration between perspectives”.There is much more to be learned about the articulation and management of governance structures and mechanisms in public health practice. Governance structures, their role in the practice of public health in Ontario, and their potential utility in facilitating holistic and systems-based approaches towards health, thus warrant further research.

3. *Making connections, forming bridges and closing gaps*The importance of relationship-building was another key theme which emerged from the interviews. Both formal and informal relationships were found at the grassroots and community level, across disciplines and knowledge cultures, across diverse governmental and non-governmental sectors, as well as internationally. Although building these partnerships required extensive time, energy, and resources, they were seen to be valuable tools to help participants overcome a number of the challenges previously discussed in this report. For example, developing strong relationships with diverse stakeholders could help to connect disparate perspectives, break down silos and develop a shared understanding among those involved.A shared understanding of holistic perspectives around health and the environment could also be cultivated through advocacy partnerships. Collaborations between various organizations could help to push particular issues or perspectives forward. For example, one participant cited a collaborative report by the World Health Organization, the Food and Agriculture Organization of the United Nations, the World Organization for Animal Health (OIE), the World Bank, UNICEF and the United Nations System for Influenza Coordination, as helping to advocate for One Health approaches towards health [12].Participants found partnerships to be essential in understanding and applying a systems lens to their work, and in promoting longer-term health outcomes and program visions.Partnerships were also seen as fundamentally important to building capacity within communities. Working closely with stakeholders and engaging community members could lead to greater empowerment and increased ability for groups to “*articulate policies and to bring them forward to key decision-makers locally*”. These community engagement strategies were also seen to be essential in the creation of sustainable health outcomes that could be replicated “*across neighbourhoods and communities*”. In fact, one participant highlighted sustainability and replicability as key components of their programming. Healthy and empowered communities could become important models of practice and sources of learning for other communities.Closely related to the theme of partnerships, the idea of connections and acting as connectors strongly resonated with some participants. For these individuals, developing these connections within their larger network of stakeholders was a core component of their mandate and identity.Developing connections was also seen as a key step towards the creation of healthy public policy. For example, one participant discussed the importance of involving a wide range of stakeholders in policy development processes. This participant cited the knowledge translation strategy of the Canadian Institutes of Health Research (CIHR) to demonstrate the important role that community members and end-users have to play in research and policy creation.Similarly participants addressed the need for closer and healthier collaborations between scientists, policy makers and the public. In particular, one participant recognized the need to develop close working relationships with key political figures and decision makers.Although participants recognized the wide range of benefits that could arise out of partnerships and collaborations, they also identified several significant challenges to overcome. For example, although including non-traditional voices in the larger discourse could provide valuable insights from alternate points of view, some participants discussed the difficulties of engaging groups who had such different perspectives on issues such as health.Additionally issues of sensitivity may be created when bringing particular types of stakeholders to the table. Overcoming this barrier may require political finesse and manoeuvring around sensitive topics. For example, one participant noted the challenge of working with industry partners on particular issues due to their financial self-interests. However, rather than censoring these voices, the participant felt that it was incumbent upon scientists and researchers to be more reflective and more forthcoming with regard to their own biases and self-interests.Finally, a lack of resources was identified as one of the biggest challenges towards developing partnerships and collaborations. Participants identified time, finances and manpower as essential requirements for these holistic working practices. For example, engaging community members, closely working with grassroots groups, and applying a systems lens to their programming, were all seen as intensive activities that required a significant investment of resources.Certainly the importance of forming partnerships and working collaboratively with stakeholders is well recognized within public health. Participants in these interviews described the immense value of forming relationships and connections with communities and stakeholders. As stated by one participant, at its core, “*public health itself is integrative*”. However, the simplistic and intuitive nature of this idea belies the immense challenges in realizing this goal. Indeed, these obstacles were a major theme which consistently emerged during the key informant interviews. As will be discussed in the next section, the difficulties of operationalizing these concepts may be one of the most significant challenges to adopting holistic practices in public health.

4. Challenging next steps: how can we operationalize EcoHealth and One Health?A number of researchers have offered up various pillars, models and heuristics to try and best explain EcoHealth and One Health. Charron [9] suggests six core principles which may be used to frame health under a holistic and systemic lens. The sixth principle, ‘knowledge-to-action’ refers to when the “knowledge gained from research is used to improve health and well-being” [9] by considering and improving the larger ecosystem.In considering the rich information emerging from all these interviews and the lessons learned from the experiences of all the participants, it is unclear as to where all this knowledge will eventually lead. Certainly, this is a reflexive issue that was not only struggled by the primary researcher of this study but also by the participants themselves. Despite recognizing the merits of holistic approaches to public health practice, some participants were challenged to in translating these ideas into tangible programs and policies for their organization.Other participants were able to identify a number of key steps that will first need to be taken in order for holistic and systems thinking to better inform the practice of public health in Ontario. These steps will require effective engagement of actors from across disciplines, organizations and knowledge cultures. There must also be change occurring at varying scales and magnitudes of the system - from the broad societal level down to the individual, from the federal government down to the local community, and from the top of a political-management structure down to the scientist.As previously discussed, greater investments of time, money and human resources are required, in order for these changes to materialize. Change can also be cultivated by greater championing of these ideas within public health. Political and organizational leaders were seen to be highly valuable in several ways. Not only could they promote a change in the way that society and organizations perceive and think about our health and well-being in the context of the larger ecosystem, but they could change the way that we think about our own roles and mandates as actors within the public health sphere.One of the biggest barriers preventing participants from broadly adopting holistic principles into their work was the lack of indicators and measures of health in the larger system. Many participants found current metrics to be inadequate for measuring and understanding broader public health issues. For example, it was recognized that traditional health outcomes such as rates of disease and illness only presented one facet of the overall health of communities. Extending participants’ discussions on holistic definitions of health, well-being and the environment, into specific and measurable markers were challenging to say the least.Additionally, there is a lack of evaluative tools to measure the success of programs that applied holistic and systems-framed approaches towards health. This became especially problematic when participants redefined their role as public health actors: not just as defenders against disease and sickness, but as capacity builders, connectors of systems, and promoters of health and well-being. In this new role, definitions and measures of success were ambiguous. How does one measure the intangibles of their work? This challenge becomes increasingly complicated when multiple stakeholders and partners are involved.In contrast, one participant seemed to find some success in transforming these broader holistic goals into specific objectives of their work. For example, issues of social equity were seen not just as lofty, feel-good ideals, but as specific targets that could be measured and evaluated within their programming.Certainly a key step in the advancement of holistic and systems thinking in public health, is to develop better ways to reflect upon and evaluate the impact that public health actors are having on their communities.Lastly, there needs to be organizational changes to the way that public health actors work with each other. As discussed previously, participants in these interviews consistently stressed the need to build working relationships across organizations, disciplines and sectors. In order for these healthy collaborations to manifest and for partners to effectively collaborate, the way that we share and integrate each other’s knowledge needs to be better articulated. Thus agreements on data sharing, data ownership, accountability and other standard operating procedures will need to be carefully constructed, as the structural foundations for these collaborative relationships. The importance of these data agreements was highlighted by one participant’s observation that:As mentioned by one participant, the concepts which form EcoHealth and One Health are intuitive and “*easy to sell*”. And as seen by some of the themes emerging from these interviews, these ideas are generally accepted by study participants and at least by some of their partners. For these participants, the greater task at hand seemed to be, ‘how do they translate this knowledge into action’?Understanding how these concepts can be transformed into tangible actions is no simple task and certainly merits much more research and reflection. In fact both the Public Health Agency of Canada [11] and the Centre for Disease Control and Prevention [21] have recently held working meetings that tried to understand how to solve this challenge: how can EcoHealth and One Health be operationalized in the public health sphere? Interestingly, many of the key themes and findings from this current study echo several of the conclusions described in the two reports arising from these meetings [11,21]. Both reports emphasize the importance of better cross-sectoral collaborations and working relationships, incorporating community and non-traditional stakeholders into partnerships, improving methods of data sharing, and harnessing political forces as a means to advocate for and push forward EcoHealth and One Health ideas.How can the ‘knowledge-to-action’ pillar of EcoHealth be achieved? How might the lessons and insights gleaned from these interviews be used to shape the practice of public health in Ontario? Simple and easy answers to these questions are lacking. However the participants in this study did identify some key steps and processes which must occur before these holistic approaches can become commonplace and standard working practices in the public health sphere.For example, one area which merits more research is the study of models of collaboration and partnerships - especially those which engage multiple sectors, perspectives and knowledge communities. Develop a clear understanding of the critical success factors of sustainable cross-sectoral partnerships could have important lessons for public health actors. One organization researching concepts in integrated governance within public health is the National Collaborating Centre for Healthy Public Policy (NCCHPP). For example, a recent NCCHPP report profiled two Canadian initiatives which centred on integrated governance – ActNow BC and Section 54 of Quebec’s Public Health Act [22]. In their preliminary analysis of these initiatives, the authors were able to compare two governance approaches used in these initiatives for developing healthy public policy: horizontal management and a whole-of-government approach. Neither of the initiatives studied by the NCCHPP specifically focus on the connection between health and the environment. Thus there is a need to identify and evaluate additional integrated governance initiatives in the area of health and the environment, and the role of strategies such as horizontal management or a whole-of-government approach in the success of these initiatives.Certainly, focusing on the gritty details of organizational structures and policies, and the processes by which public health work is performed, seems far removed from the broad issues of health within a larger ecosystem. However, as underscored by the previous discussions, tackling these next steps are necessary tasks before public health can move towards a practice that is integrative, holistic and inclusive of systems perspectives.

### Study limitations

Although the ten interviews provided some rich insights and important lessons around EcoHealth and One Health in public health practice in Ontario, the limited size and scope of the study should be taken into consideration. A more comprehensive study would engage additional perspectives across the broad public health sphere. A larger sample size could also allow for further analysis such as making thematic comparisons between participants from different levels of government, or between different areas within public health. Finally, a larger study with multiple coders could calculate measures such as intercoder agreement to increase the study’s validity [23]. Given the limited sample size, the findings of this study are not meant to be generalized across the entire public health sphere in Ontario. Rather the aim of this study was to explore lessons and insights from the experiences of some public health actors in Ontario, with regards to holistic and integrative approaches connecting health and the environment.

## Conclusions

One Health and EcoHealth are still relatively unknown within the public health sphere. Indeed during the recruitment process, it was noted that many individuals (including the study participants) were either completely unfamiliar or had only a vague understanding of these approaches. However, during these interviews, the study participants discussed and reflected upon ideas heavily couched within One Health and EcoHealth thinking.

Participants approached the concepts of health and the environment through multiple lenses, and also viewed them as shifting and contextual ideas. They spoke of the strong multi-dimensional connections between health and the larger ecosystem, and talked about ways that these connections were manifested in their policies and programming. Participants spoke of concepts such as equity, sustainability, and considering the larger system involved in their work. Interestingly, these key ideas and values are also enshrined as core principles of EcoHealth work [9].

Along with the strong shades of One Health and EcoHealth thinking which emerged from the interviews, there were also a number of important discussions around barriers and support systems for these approaches. For example, governance and models of collaboration were identified as key factors that could either hinder or facilitate holistic and integrative public health practices. In order for public health actors to work collaboratively within systems, a number of structural and organizational processes such as data sharing agreements will need to be developed. Although many participants highly valued the perspectives offered by systems approaches, participants greatly emphasized the need to develop indicators or measures of success when working in systems.

Both One Health and EcoHealth have received a host of impressive accolades and endorsements, for offering a unique understanding of health: one that is pluralistic, equitable and sustainable; one that engages individuals and organizations across sectors, disciplines and knowledge cultures; and one which promises to improve not only the health of people but also of the larger ecosystem in which they live in. Importantly, both these approaches have also received their fair share of criticisms as well. Skeptics often attack One Health and EcoHealth for demanding too much: their goals are too idealistic and too lofty, their resource requirements are too costly, and their expectations of successful cross-sectoral partnerships are too high. In an era of government cutbacks and budgetary cost-cutting, and in a field where siloed thinking is far too common, these are important concerns to consider.

Still, there seems to be hope for these types of ideas in public health. Despite the limited scope of the study, the rich data from all the interviews seem to indicate that there is an inclusive space, for at least some aspects of One Health and EcoHealth to flourish. Within academia and among the EcoHealth and One Health communities, there is often much debate as to the scope and boundaries of each approach. Given the strong similarities in their philosophies and principles, clearly articulating where one approach ends and the other begins is challenging. Certainly there is merit in better defining EcoHealth and One Health, and how they can complement each other in understanding issues at the human-animal-environment interface. This may be especially important both for pedagogical purposes and for increasing awareness among public health actors, of the value of these approaches in tackling complex public health issues.

In this study, many of the participants lacked a strong familiarity with One Health or EcoHealth. But as previously described, the participants’ thoughts, values and experiences were heavily imbued with core principles of these approaches. This may indicate that One Health and EcoHealth concepts may themselves be ‘emergent properties’ within the public health sphere. In other words, in the absence of academia espousing these principles or governments mandating these ‘new’ approaches to public health practice, these ideas may have already germinated of their own accord. It is unclear how these ideas may grow and develop within the next stage of their journey but no doubt, they will have strong implications for the future practice of public health.

## Competing interests

The authors declare that they have no competing interests.

## Authors’ contributions

ZL participated in the study design, conducted the study, performed the coding and content analysis, and drafted the manuscript. DM helped to conceive the study, participated in the study design and helped to review the manuscript. KM helped to conceive the study, participated in the study design and helped to review the manuscript. All authors read and approved the final manuscript.

## Pre-publication history

The pre-publication history for this paper can be accessed here:

http://www.biomedcentral.com/1471-2458/12/358/prepub
